# Characterization of *Escherichia coli* virulence genes, pathotypes and antibiotic resistance properties in diarrheic calves in Iran

**DOI:** 10.1186/0717-6287-47-28

**Published:** 2014-06-23

**Authors:** Masoud Shahrani, Farhad Safarpoor Dehkordi, Hassan Momtaz

**Affiliations:** Young Researchers and Elites Club, Shahrekord Branch, Islamic Azad University, Shahrekord, Iran; Department of Microbiology, College of Veterinary Medicine, Shahrekord Branch, Islamic Azad University, P.O. Box: 166, Shahrekord, Iran

**Keywords:** Antibiotic resistance profile, Diarrheic calves, *Escherichia coli*, Iran, Serogroups, Virulence genes

## Abstract

**Background:**

Calf diarrhea is a major economic concern in bovine industry all around the world. This study was carried out in order to investigate distribution of virulence genes, pathotypes, serogroups and antibiotic resistance properties of *Escherichia coli* isolated from diarrheic calves.

**Results:**

Totally, 76.45% of 824 diarrheic fecal samples collected from Isfahan, Chaharmahal, Fars and Khuzestan provinces, Iran were positive for *E. coli* and all of them were also positive for *cnf2, hlyA, cdtIII, f17c*, *lt, st*, *stx1, eae, ehly*, *stx2* and *cnf1* virulence genes. Chaharmahal had the highest prevalence of STEC (84.61%), while Isfahan had the lowest (71.95%). *E. coli* serogroups had the highest frequency in 1–7 days old calves and winter season. Distribution of ETEC, EHEC, AEEC and NTEC pathotypes among *E. coli* isolates were 28.41%, 5.07%, 29.52% and 3.49%, respectively. Statistical analyses were significant for presence of bacteria between various seasons and ages. All isolates had the high resistance to penicillin (100%), streptomycin (98.25%) and tetracycline (98.09%) antibiotics. The most commonly detected resistance genes were *aadA1*, *sul1*, *aac*[3]*-IV, CITM*, and *dfrA1*. The most prevalent serogroup among STEC was O26.

**Conclusions:**

Our findings should raise awareness about antibiotic resistance in diarrheic calves in Iran. Clinicians should exercise caution when prescribing antibiotics.

## Background

Calf diarrhea is one of the most economic and pervasive concern in veterinary industry all around the world. Infectious agents are the most commonly detected causes of calf diarrhea [1–3]. Several studies have been addressed the high distribution of *Escherichia coli* (*E. coli*) strains in infectious calf diarrhea [3–6]. *Escherichia coli* is a gram-negative, rod-shaped, flagellated, non-sporulating and facultative anaerobic bacterium of the family enterobacteriaceae that classically classified into enterohemorrhagic (EHEC), enterotoxigenic (ETEC), necrotoxigenic (NTEC), enteroinvasive (EIEC), enteropathogenic (EPEC) and attaching and effacing *E. coli* (AEEC) pathotypes [7]. Intimin genes are present in EPEC and some Shigatoxin-producing *E. coli* (STEC). EPEC strains are defined as *eae* harbouring diarrhoeagenic *E. coli* that possess the ability to form attaching-effacing (A/E) lesions on intestinal cells and that do not possess Shigatoxin encoding genes [8, 9]. Diarrheagenic *Escherichia coli* are now broadly placed into 6 classes based on virulence mechanisms. One of these classes, enterotoxigenic *E. coli*, is the most common cause of diarrhea in beef and dairy calves in the first 4 days of life. Two other diarrheagenic classes, EHEC and EPEC, are important causes of disease in human beings, but less well substantiated causes of diarrhea in calves [8, 9]. *E. coli* strains that cause hemorrhagic colitis and hemolytic uremic syndrome in humans, express high levels of Shiga toxin, cause A/E lesions in intestinal epithelial cells, and possess a specific 60-MDa EHEC plasmid are known as EHEC [8, 9]. One feature EHEC and EPEC have in common is the causation of intestinal epithelial lesions known as A/E. AEEC is a designation for those *E. coli* strains known to cause A/E lesions or at least carry the genes for this trait, and therefore include organisms that fall into either the EHEC or EPEC classes. Because cattle are carriers of many different serotypes of EHEC, much emphasis has been placed on the public health and food safety concerns associated with the fecal shedding of these organisms. However, much less emphasis has been given to their roles as diarrheagenic pathogens of cattle [8, 9]. In fact, several pathotypes of *E. coli* have been known by presence of certain genes. *EAF* and *bfp* are predominant in EPEC and typical genes of EAEC (*pAA*), *cdt* and *cnf* are the major genes of NTEC and finally *sta, stb, lt, f4, f5, f18, stx1* and szx*2* are the genes of the EHEC pathotype [8, 9].

EHEC strains are a subset of STEC strains [3, 7]. Infection with STEC strains can result in a spectrum of outcomes, ranging from asymptomatic carriage to uncomplicated diarrhea, bloody diarrhea, hemolytic uremic syndrome (HUS), thrombocytopenia, hemolytic anemia, and acute renal failure [3, 7, 10]. Most outbreaks and sporadic cases of bloody and non-bloody diarrhea and HUS have been attributed to strains of the STEC serogroups including O157, O26, O103, O111, O145, O45, O91, O113, O121 and O128 [11–15].

Heat-labile enterotoxins (LT) and heat-stable enterotoxins (STa or STb) are two of the most important bacterial virulence factors are able to causes severe diarrhea in calves [3, 10]. Also, there are some another virulence factors including phage-encoded cytotoxins, called Shiga toxin 1 (*stx1*) and Shiga toxin 2 (*stx2*), the protein intimin (*eae*) and the plasmid-encoded enterohaemolysin or enterohaemorrhagic *E. coli* haemolysin (*ehly*) which are related to the pathogenesis of STEC strains [16]. The NTEC are able to elaborate two types of cytotoxic necrotizing factors (CNF1 and CNF2). CNF factors are heat-labile proteins which can cause diarrhea. Cytolethal distending toxin (CDT) induces enlargement and death of some cultured eukaryotic cell lines and causing diarrhea. Totally, five different *cdt* alleles (*cdt-I*, *cdt-II*, *cdt-III*, *cdt-IV* and *cdt-V*) have been reported in *E. coli* strains [17–21]. The 31A produces F17c fimbria (formerly called 20 K), which is responsible for *N*-acetyl-D-glucosamine-dependent adhesion of bacteria to calves border villi [5, 22]. The F17c includes fimbriae expressed by calf diarrheic [23, 24]. Studies in France, Scotland and Belgium showed that pathogenic F17-producing *E. coli* strains represent a significant part of the bacterial strains isolated from diarrheic calves [25].

Diseases caused by *E. coli* often require antimicrobial therapy; however, antibiotic-resistant strains of this bacterium cause longer and more severe illnesses than their antibiotic-susceptible counterparts. Several studies have shown that antibiotic resistance in *E. coli* has increased over time [26–28]. Data on the distribution of serogroups, pathotypes, virulence genes and the antimicrobial resistance properties of *E. coli* strains isolated from diarrheic calves is scarce in Iran. Therefore, the aim of the present study was to characterize *E. coli* strains isolated from Iranian diarrheic calves at the molecule level and investigate their susceptibility to 13 commonly used antibiotics, as well as investigating seasonal variation in the prevalence and serogroup distribution of *E. coli*.

## Results and discussion

We found that 630 out of 824 samples (76.45%) were positive for *E. coli*. Chaharmahal province had the highest incidence of *E. coli* in diarrhea specimens (84.61%), while Isfahan province had the lowest incidence (71.95%).

Distribution of various pathotypes is shown in Table 1. We found that the incidence of ETEC, EHEC, AEEC and NTEC pathotypes were 28.41%, 5.07%, 29.52% and 3.49%, respectively. In addition, 33.49% diarrheic samples were diagnosed as non detected. We found significant differences between the incidence of ETEC and NTEC (*P* = 0.017), ETEC and EHEC (*P* = 0.023), AEEC and NTEC (*P* = 0.015) and AEEC and EHEC (*P* = 0.021) pathotypes of *E. coli. Lt* and *K99* (F5) genes were detected in all of the ETEC pathotype, while the *st* gene was detected in 4.46% of them. *F41* gene was detected in 72 samples of the EHEC pathotype (40.22%), included all *stx1, eae, ehly* genes, while the distribution of *stx1*, *stx2* and *eae* genes in AEEC pathotype were 96.23%, 56.98% and 51.07%, respectively. In addition, 91 samples (48.92%) had both *stx1* and *eae*, 61 (32.79%) samples had both *stx2* and *eae* and 34 (18.27%) had all *stx1, stx2* and *eae* genes. Furthermore, both *cnf1* and *hlyA* genes were detected in 14 (63.63%), both *cnf2* and *hlyA* genes in 6 (27.27%), all *cnf1, cnf2* and *hlyA* genes in 4 (18.18%), all *cnf1, cnf2, hlyA* and *cdtIII* genes in 4 (18.18%), all *cnf1, hlyA* and *f17c* genes in 2 (9.09%), all *nf2, hlyA* and *f17c* genes in 2 (9.09%), all *cnf2, hlyA and cdtIII* genes in 20 (90.90%) and finally, all *cnf2, hlyA, cdtIII* and *f17c* genes in 5 (22.72%) of NTEC pathotypes (Table 1). We also found significant differences between the incidences of *lt* and *st* (*P* = 0.033), *f5* and *f41* (*P* = 0.048), *stx1* and *stx2* (*P* = 0.046) and *stx1* and *eae* (*P* = 0.038) genes.

**Table 1:** **Prevalence of virulence genes in** ***E. coli*** **pathotypes isolated from diarrheic calves**

Pathotype	No. positive samples	Virulence gene
Non detected	211 (33.49%)	-
ETEC	179 (28.41%) A, B*	*lt, F5*: 179 (100%) A, B
		*lt, st*, F5: 8(4.46%) a
		*F5, F41*: 72 (40.22%) b
		*F5, lt*: 179 (100%)
		*F41, lt*: 67 (37.43%)
		*F41, st, lt*: 4 (2.23%)
EHEC	32 (5.07%) a, c	*stx1, eae, ehly*: 32 (100%)
AEEC	186 (29.52%) C, D	*stx1*: 179 (96.23%) A
		*stx2*: 106 (56.98%) a
		*eae*: 95 (51.07%) a
		*stx1, ea*e: 91 (48.92%)
		*stx2, eae*: 61 (32.79%)
		*stx1, stx2, eae*: 34 (18.27%)
NTEC	22 (3.49%) b, d	*cnf1, hlyA*: 14 (63.63%)
		*cnf2, hlyA*: 6 (27.27%)
		*cnf1, cnf2, hlyA*: 4 (18.18%)
		*cnf1, cnf2, hlyA, cdtlll*: 4 (18.18%)
		*cnf1, hlyA, f17c*: 2 (9.09%)
		*cnf2, hlyA, f17c*: 2 (9.09%)
		*cnf2, hlyA, cdtlll*: 20 (90.90%)
		*cnf2, hlyA, cdtlll, f17c*: 5 (22.72%)
Total	630 (76.45%)	

*Similar capital and small letters in column have a significant differences about *P* <0.05.

Seasonal distribution of *E. coli* pathotypes is shown in Table 2. Samples that were collected in the winter had the highest incidence of bacteria (54.92%), while those were collected in summer had the lowest incidence (2.53%). Our results showed significant differences (*P* = 0.018) for presences of *E. coli* strains between cold and warm seasons. The age distribution of the diarrheic calves with regard to infection with *E. coli* is shown in Table 3. We found that the 1–7 day-old calves had the highest incidence of *E. coli* (39.19%), while the 22–30 day-old calves had the lowest incidence (12.01%). Statistical analysis were significant for the incidence of *E. coli* strains between younger and older calves (*P* = 0.035 between 1–7 days old and 15–21 and *P* = 0.026 between 1–7 days old and 22–30).

**Table 2:** **Prevalence of** ***E. coli*** **pathotypes isolated from diarrheic calves in different seasons and provinces**

Province	Season	Non detected	ETEC	STEC	NTEC	No. pos. samples
Isfahan (246)	Autumn (84)	18	7	21	-	46
	Winter (111) A*	39	53	1	9	102
	Spring (35)	7	7	11	1	26
	Summer (16) a	1	-	2	-	3
Chaharmahal (182)	Autumn (61) C	23	9	18	3	53
	Winter (95) B	37	42	11	1	91
	Spring (17)	1	1	-	1	3
	Summer (9) b, c	2	1	4	-	7
Fars (208)	Autumn (84) E	22	6	33	2	63
	Winter (94) D	22	10	35	2	69
	Spring (20)	5	16	1	-	22
	Summer (10) d, e	-	3	-	-	3
Khozestan (188)	Autumn (63) G	5	1	25	3	34
	Winter (86) F	23	13	48	-	84
	Spring (30)	5	8	8	-	21
	Summer (9) f, g	1	2	-	-	3
Total (824)	Autumn (292) C	68	23	97	8	196
	Winter (386) H	121	118	105	12	346
	Spring (102)	28	32	10	2	72
	Summer (44) h, i	4	6	6	-	16

*Similar capital and small letters in column have a significant differences about *P* <0.05.

**Table 3:** **Prevalence of** ***E. coli*** **pathotypes isolated from diarrheic calves in different ages and provinces**

Province	Age (day)	Non detected	ETEC	STEC	NTEC	No. pos. samples
Isfahan (246)	1-7 A*	36	42	10	-	88
	8-14	14	6	18	1	39
	15-21 a	1	3	23	2	29
	22-30 a	1	2	14	4	21
Chaharmahal (182)	1-7 B	33	29	9	-	71
	8-14	10	6	18	2	36
	15-21 b	1	2	18	3	24
	22-30 b	1	1	17	4	23
Fars (208)	1-7 C	38	37	7	-	82
	8-14 D	21	5	10	-	36
	15-21 c,d	1	4	17	1	23
	22-30 c,d	2	1	12	1	16
Khozestan (188)	1-7 E	36	36	6	-	78
	8-14	15	3	13	1	32
	15-21 e	1	1	10	1	13
	22-30 e	-	1	16	2	19
Total (824)	1-7 F	143	144	32	-	319
	8-14	60	20	59	4	143
	15-21 f	4	10	68	7	89
	22-30 f	4	5	59	11	79

*Similar capital and small letters in column have a significant differences about *P* < 0.05.

Antimicrobial resistance of *E. coli* pathotypes isolated from the diarrheic calves is shown in Table 4. Bacterial strains exhibited the highest level of resistance to penicillin (100%), followed by streptomycin (98.25%), tetracycline (98.09%), lincomycin (92.69%) and sulfamethoxazol (90.31%), while STEC and ETEC had the highest resistance profiles overall. Totally, the *E. coli* strains of our investigation had the lowest resistance to nitrofurantoin (23.96%) and cephalothin (52.06%). There were significant differences between resistance of *E. coli* strains to penicillin and nitrofurantoin (*P* = 0.013), penicillin and cephalothin (*P* = 0.045), streptomycin and nitrofurantoin (*P* = 0.015), streptomycin and cephalothin (*P* = 0.050), tetracycline and nitrofurantoin (*P* = 0.018), lincomycin and nitrofurantoin (*P* = 0.026) and sulfamethoxazol and nitrofurantoin (*P* = 0.029).

**Table 4:** **Antibiotic resistance profile of** ***E. coli*** **pathotypes isolated from diarrheic calves (Disk diffusion method)**

	P10*	TE30	S10	C30	SXT	GM10	NFX5	L2	CF30	CIP5	TMP5	F/M300	AM10
Non detected (211)	211	209	210	145	201	179	161	196	102	112	189	32	126
ETEC (179)	179	179	175	126	152	150	101	169	118	120	72	18	116
STEC (218)	218	208	214	192	198	167	123	201	106	146	123	96	202
NTEC (22)	22	22	20	2	8	6	2	18	2	2	8	5	4
Total (630)	630 (100%) A**	618 (98.09%) C	619 (98.25%) B	465 (73.80%)	559 (90.31%) E	502 (79.68%)	387 (61.42%)	584 (92.69%)	328 (52.06%) a,b	380 (60.31%)	392 (62.22%)	151 (23.96%) a,b,c,d,e	448 (71.11%)

*In this table P10 = penicillin (10 μ/disk); TE30 = tetracycline (30 μg/disk); S10 = streptomycin (10 μg/disk); C30 = chloramphenicol (30 μg/disk); SXT = sulfamethoxazol (25 μg/disk); GM10 = gentamycin (10 μg/disk); NFX5 = enrofloxacin (5 μg/disk); L2 = lincomycin (2 μg/disk); CF30 = cephalothin (30 μg/disk); CIP5 = ciprofloxacin (5 μg/disk); TMP5 = trimethoprim (5 μg/disk); F/M300 = nitrofurantoin (300 μg/disk); AM10 = ampicillin (10 μ/disk).

**Similar capital and small letters in rows have a significant differences about *P* <0.05.

The distribution of antimicrobial resistance genes within the bacterial pathotypes isolated from diarrheic calves is shown in Table 5. Genes that encode resistance to streptomycin, sulfonamide, gentamicin, ampicillin and trimethoprim antibiotics, i.e., *aadA1*, *sul1*, *aac*[3]*-IV*, *CITM* and *dfrA1* were the most common antibiotic resistance genes in the diarrheic calves. Interestingly, we found that ETEC and STEC had the highest frequency of antibiotic resistance genes. Statistical analyses were significant between the incidence of *aadA1* and *cmlA* (*P* = 0.018), *aadA1* and *qnr* (*P* = 0.024), *aadA1* and *cat1* (*P* = 0.028), *sul1* and *cmlA* (*P* = 0.019), *sul1* and *cat1* (*P* = 0.029) and *sul1* and *qnr* (*P* = 0.031) antibiotic resistance genes.

**Table 5:** **Distribution of antibiotic resistance genes of** ***E. coli*** **pathotypes isolated from diarrheic calves in different provinces**

	Province	*aadA1*	*tetA*	*tetB*	*dfrA1*	*qnr*	*aac* *(3)* *-IV*	*sul1*	*blaSHV*	*CITM*	*cat1*	*cmlA*
Non detected (211)	Isfahan (63)	57	23	16	46	10	61	56	49	54	15	7
	Chaharmahal (59)	58	18	22	49	14	56	59	11	48	10	3
	Fars (44)	38	21	16	38	2	40	42	9	41	-	10
	Khozestan (45)	43	37	37	35	3	13	41	12	43	5	4
ETEC (179)	Isfahan (46)	44	36	13	18	5	45	42	28	31	20	7
	Chaharmahal (36)	34	22	19	17	6	32	29	26	30	26	6
	Fars (44)	39	30	14	14	2	29	32	25	31	13	7
	Khozestan (53)	52	14	17	14	3	36	41	13	9	7	6
SETC (218)	Isfahan (62)	58	30	32	36	32	43	52	20	49	14	-
	Chaharmahal (58)	55	28	26	29	36	49	56	29	52	16	36
	Fars (59)	56	14	16	32	16	46	51	29	55	-	40
	Khozestan (39)	33	26	24	23	17	31	33	8	36	2	18
NTEC (22)	Isfahan (6)	4	2	1	3	2	1	8	1	3	-	1
	Chaharmahal (1) a*	1	-	1	1	-	-	1	-	1	-	-
	Fars (10) A	10	6	-	1	3	1	3	-	1	-	-
	Khozestan (5)	2	2	2	1	1	1	2	-	1	-	-
Total	Isfahan (177) A	163A	91	62	103	49a,b	150	158B	98	137	49a,b	15a,b
	Chaharmahal (154)	148C	68	68	96	56c	137	145	66	131	52c	45
	Fars (157)	143D	71	46	85	23d	116	128	63	128	13d	50
	Khozestan (142) a	130E	79	80	73	24e	81	117	33	89	14e	28

*Similar capital and small letters in rows have a significant differences about *P* <0.05.

Incidence of STEC O-serogroups in diarrheic calves is shown in Table 6. O26 (26.60%) had the highest incidence, followed by O157 (14.67%). In addition, the serogroups of 19 samples (8.71%) cannot be detected. The results of our study showed significant difference between the presence of O26 and O113 (*P* = 0.014), O26 and O121 (*P* = 0.016), O26 and O45 (*P* = 0.020), O26 and O128 (*P* = 0.022), O26 and O145 (*P* = 0.027), O157 and O113 (*P* = 0.018) O157 and O121 (*P* = 0.021) and O157 and O45 (*P* = 0.025) serogroup in calves with diarrhea. The distribution of virulence factors, O-serogroups and antibiotic resistance genes in non-pathogenic *E. coli* strains were lower than those of pathogenic strains. Also, non-pathogenic strains of bacteria were more susceptible to tested antibiotics than pathogenic bacteria.

**Table 6:** **Prevalence of STEC serogroups isolated from diarrheic calves in different provinces**

Province	0157	026	0103	0111	0145	045	091	0113	0121	0128	Non detected
Isfahan (62)	16	16	-	3	4	2	8	6	-	4	3
Chaharmahal (58)	2	-	6	7	8	10	10	-	3	6	6
Fars (59)	10	28	9	-	2	-	6	-	-	-	4
Khozestan (39)	4	14	-	6	-	-	2	-	4	3	6
Total (218)	32B*	58A	15	16	14a	12a,b	26	6a,b	7a,b	13a	19

*Similar capital and small letters in rows have a significant differences about *P* <0.05.

The high importance of geographical area and season on the incidence of *E. coli* strains in diarrheic calves were addressed in the present study. Similar results have been reported by Mohamed Ou Said et al. [29]. Season and geography are also known to influence passive transfer of colostral immunoglobulins in calves [30]. Season has a significant effect on the calf mortality [31] as well as on the absorption of immunoglobulins in neonatal calves. One possible explanation for the high prevalence of *E. coli* strains in calves in winter is that climatic variables such as heat, rain and thunderstorms, together with variable barometric pressure may have affected the autonomic nervous systems. These variables could affect immunity, thus making calves more susceptible to infections. Alternatively, the higher prevalence of *E. coli* strains in winter in our study may be related to the fact that the mean serum IgG1 concentrations were low in winter born calves and increased during the spring and summer [32]. The higher mortality rates of 69.6% and 15.36% were observed in winter born calves than 39.4% and 5.97% in summer born calves of Afzal et al. [33] and Sharma et al. [34] investigations. Similar results have been reported previously [35, 36].

To the best of our knowledge, this is the first and most comprehensive report on serogroups, pathotypes, virulence genes and antimicrobial resistance properties in *E. coli* strains isolated from diarrheic calves in Iran. Totally, the prevalence of *E. coli* strains isolated from calves with diarrhea in Iran (76.45%, our results) was significantly higher than Egypt (10.36%) [37] and India (42.65%). Mora et al. [38] reported that 12% of the calves and 22% of the farms samples were positive for highly virulent STEC serotype O157:H7. Unfortunately, there were limited investigations related to distribution of *E. coli* bacterium and its pathotypes, serogroups, virulence factors and antibiotic resistance properties.

Another important finding relates to the distributions of several bacterial virulence factors in the diarrheic calves of our investigation. A Brazilian study [39] showed that 49.75% of *E. coli*-positive diarrheic samples was toxigenic and the most prevalent virulence genes were *stx1* (9.7%), *stx2* (6.3%), α-hemolysin (9.7%), *ehly* (6.8%), *cnf1* (0.5%), *LT-II* (8.3%) and *STa* (3.9%). Nguyen et al. [3] reported that 31.30% of *E. coli* strains of diarrheic calves had one of the fimbrial genes. They revealed that the incidence of shiga toxin genes, enterotoxins and *eae* gene were 46.29%, 0.92%, and 31.48%, respectively.

Herrera-Luna et al. [40] revealed that 17% of all diarrheic and healthy calves of Australian herds were infected by *E. coli*. They showed that 15.2% of *E. coli* strains harbored the shiga toxin genes including *stx1, stx2* and *ehly* and *eae* genes. Low incidence of VTEC phenotype and O157:H7 serotypes of *E. coli* strains of diarrheic calves of Najaf, Iraq were reported by Al-Charrakh and Al-Muhana [41]. Diarrheic calves of Bradford et al. [42] investigation hadn’t any *cnf1*, cnf2, *stx2*, *stB* and *lt* genes, while the *K99* fimbriae, *stA* enterotoxin, *stx1* and *eae* genes were detected in 8, 8, 1 and 1 isolates, respectively which was lower than our result. It seems that *stx1*, *stx2*, *eae, ehly*, *hlyA, lt, st*, *cnf1*, *cnf2, cdtIII* and *f17c* virulence genes are predominant in *E. coli* strains isolated from calves with diarrhea.

In the present study, 211 *E. coli* strains that were isolated from diarrheic samples hadn’t any virulence factors. One possible explanation for this finding is the fact that maybe these strains were non-pathogenic *E. coli* and the animals have diarrhea caused by some other infectious agent. Achá et al. [43] reported that 76% of calves were infected with *E. coli*, while the prevalence of other causative agents including *Salmonella* species and *Campylobacter* species were 2% and 11%, respectively. They also reported that 22/55 (40%) strains from diarrheal calves and 14/88 (16%) strains from healthy calves carried the K99 adhesin (*P* = 0.001).

Our results showed that O26 and O157 were the most common serogroups of our study, while O113 and O121 were the less common. Saridakis et al. [44] showed that O26, O114 and O119 were the most prevalent serogroups in *E. coli* strains isolated from diarrheic calves. Mora et al. [38] showed that 52% of *E. coli* strains isolated from bovine herds were belonged to O26, O22, O77, O4, O105, O20, O157, O113, and O171 serogroups which was similar to our results.

Bloody diarrhea, non-bloody diarrhea, HUS and other clinical complications of infection with STEC strains are serious among calves, compelling clinicians to consider the provision of early, and empirical antibiotic therapy. However, current recommendations and the available data (although limited in scope and only formally studied for O157 and non-O157-related infections in calves) suggest that antibiotics should be withheld if STEC infection is suspected, given concerns that antibiotics may trigger release of *stx* and progression to diarrhea, resulting in worse clinical outcomes. Furthermore, because inappropriate prescriptions of antibiotics select antibiotic resistance, it was not surprising that our study found that resistance to some antibiotic agents was higher than 80%. The *E. coli* strains of our study were resistant to penicillin (100%), streptomycin (98.25%), tetracycline (98.09%), lincomycin (92.69%), sulfamethoxazol (90.31%), gentamycin (79.68%), chloramphenicol (73.8%), ampicillin (71.11%), trimethoprim (62.22%), enrofloxacin (61.42%) and ciprofloxacin (60.31%). In terms of antibiotic resistance genes, *aadA1*, *sul1*, *aac*[3]*-IV*, *CITM* and *dfrA1* were the most commonly detected.

Totally, 73.8% of the STEC strains of our study were resistance to chloramphenicol. Chloramphenicol is a banned antibiotic and the high antibiotic resistance to this drug detected in our study indicates that irregular and unauthorized use of it may have occurred in Iran. Unfortunately, veterinarians in many fields of veterinary such as large animal internal medicine, poultry and even aquaculture, use this antibiotic as a basic one. Therefore, in a short period of time, antibiotic resistance will appear. Previous study showed that in some countries 300,000 kg of antibiotics is used yearly on veterinary prescription in animals [45].

Multidrug resistance of *E. coli* strains have been reported previously [46, 47]. Rigobelo et al. [47] reported that the *E. coli* strains had the highest resistance to cephalothin (46.1%), followed by tetracycline (45.7%), trimethoprim-sulfadiazine (43.3%) and ampicilin (41.0%). Multiple resistances of *E. coli* isolates to beta-lactams antibiotics including expanded-spectrum aminoglycosides, cephalosporins, tetracycline sulphonamides, and fluoroquinolones have been reported previously [42]. De Verdier et al. [48] showed that the *E. coli* strains were resistant to sulphonamide, streptomycin, ampicillin and tetracycline. They reported that 61% of all strains were resistant to more than one antibiotic. Similar investigations have been reported from Sweden [48], United State [49] and Czech [50].

The above data highlight large differences in the prevalence of STEC strains in the different studies, as well as differences in virulence genes and antibiotic resistance properties in the clinical samples. This could be related to differences in the type of samples tested, number of samples, method of sampling, experimental methodology, geographical area, antibiotic prescription preference among clinicians, antibiotic availability, and climate differences in the areas where the samples were collected, which would have differed between each study.

## Conclusion

In conclusion, we identified a large number of pathotypes, serogroups, virulence factors and antibiotic resistance genes and resistance to more than one antibiotic in the *E. coli* strains isolated from diarrheic calves in Iran. Our data indicate that O26 and O157 serogroups are predominant in Iranian diarrheic calves. Marked seasonal, senile and geographical variation was also found. Our data revealed that the O26 serogroup, the *lt*, *f5*, *f41*, stx1, *stx2*, *eaeA* and *hlyA* putative virulence genes, the *aadA1*, *sul1*, *aac*[3]*-IV*, *CITM* and *dfrA1* antibiotic resistance genes, and resistance to penicillin, streptomycin, tetracycline, lincomycin, sulfamethoxazol, gentamycin, chloramphenicol, ampicillin, trimethoprim, enrofloxacin and ciprofloxacin were the most commonly detected characteristics of *E. coli* strains isolated from Iranian diarrheic calves. Hence, judicious use of antibiotics is required by clinicians. We suggested use of disk diffusion method.

## Methods

### Samples and *E. coli*identification

**Figure 1:**
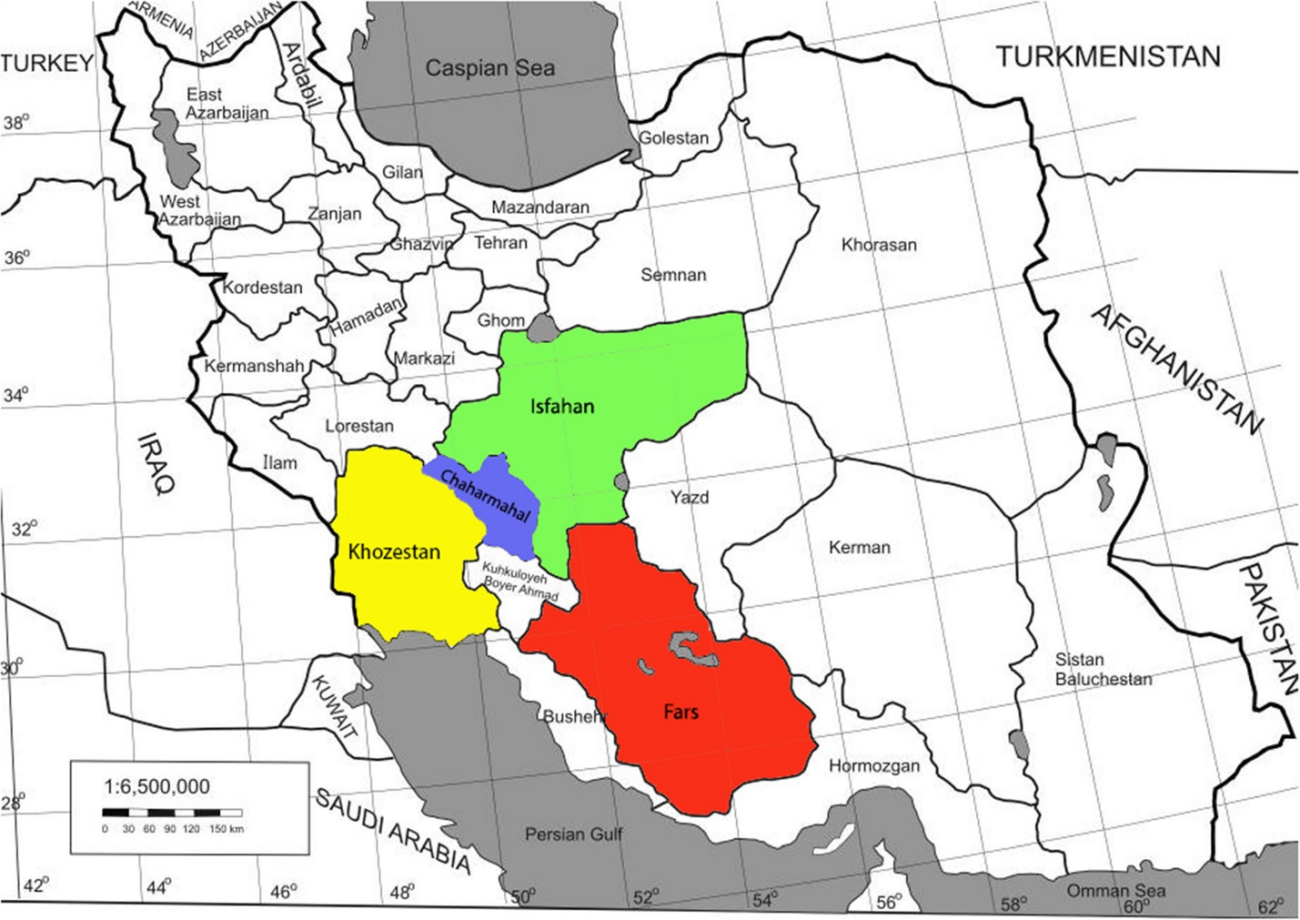
**Geographical areas of sample collection.**

Totally, 824 fecal samples of diarrheic calves were collected randomly during January 2010 to January 2011. Geographical distribution (Isfahan, Chaharmahal, Fars and Khuzestan provinces) (Figure 1), season of samples collection (spring, summer, autumn and winter) and age of diarrheic calves (2 to 30 days) were recorded during sampling. Fecal samples were taken using sterile rectal swabs. All swab samples were placed into tubes containing Stuart medium (Merck, Germany). Samples were immediately transferred to the Microbiology and Infectious Diseases Research Center of the Islamic Azad University of Shahrekord. All samples were diluted in phosphate buffered saline (PBS, Merck, Germany). Then samples were cultured on MacConkey’s agar (MC, Merck, Germany) (24 h at 37°C). Lactose positive colonies were cultured on Eosin Methylene Blue (EMB, Merck, Germany) (24 h at 37°C). Metallic green colonies were considered as *E. coli*. Several biochemical tests including Triple Sugar Iron Agar (TSI), Indole, Citrate utilization, Voges-Proskauer, urease and Methyl red tests were used for *E. coli* confirmation. All isolates were stored at -70°C in lactose broth (Merck, Germany) restraining 20% glycerol for further description.

### DNA extraction

All *E. coli* isolates were cultured on Peptone Water (PW, Merck, Germany) (24 h at 37°C). One hundred and fifty microliter of cultured PW media was added to 400 μL sterile distilled water. All components were boiled for 12 min. The mentioned suspension was frozen and then centrifuged at at 14,000 rpm for 14 min [51].

### Detection of virulence genes, antibiotic resistance genes and serogroups using Polymerase Chain Reaction (PCR)

The method of Sabat et al. [52] was used in order to *E. coli* confirmation. Several PCR protocols were used to study the presence of virulence genes, serogroups and antibiotic resistance properties of STEC strains. List of primers used for this instance is shown in Table 7. PCR conditions including temperature and volume of each reaction is shown in Table 8. All reactions were performed using the PCR thermocycler (Eppendrof Mastercycler 5330; Eppendorf-Nethel-Hinz GmbH, Hamburg, Germany). Amplified PCR products were electrophoresed on 1.5% agarose gel stained with ethidium bromide. Final products were examined using the gen documentation system. Distilled water and *E. coli* CAPM 5933, CAPM 6006, O159:H20 and O157:K88ac:H19 were used as negative and positive controls, respectively.

**Table 7:** **Oligonucleotid primers used for detection of virulence factors, serogroups and antibiotic resistance genes in STEC strains isolated from calves with diarrhea**

Primer name	Sequence (5’-3’)	Size of product (bp)	Target gene	References
**Hly-F**	GAGCGAGCTAAGCAGCTTG	889	*hlyEHEC*	[6]
**Hly-R**	CCTGCTCCAGAATAAACCACA			
**Stx1-F**	CAGTTAATGTGGTGGCGAAGG	348	*Stx1*	[53]
**Stx1-R**	CACCAGACAATGTAACCGCTG			
**Stx2-F**	ATCCTATTCCCGGGAGTTTACG	584	*Stx2*	[53]
**Stx2-R**	GCGTCATCGTATACACAGGAGC			
**Eae-F**	TGCGGCACAACAGGCGGCGA	629	*eae*	[6]
**Eae-R**	CGGTCGCCGCACCAGGATTC			
**Cnf1-F**	GGGGGAAGTACAGAAGAATTA	1111	*cnf1*	[21]
**Cnf1-R**	TTGCCGTCCACTCTCTCACCAGT			
**Cnf2-F**	TATCATACGGCAGGAGGAAGCACC	1240	*cnf2*	[21]
**Cnf2-R**	GTCACAATAGACAATAATTTTCCG			
**Cdt3-F**	GAAAATAAATGGAATATAAATGTCCG	555	*cdt-III*	[21]
**Cdt3-R**	TTTGTGTCGGTGCAGCAGGGAAAA			
**F17c-F**	GCAGGAACCGCTCCCTTGGC	416	*f17c*	[23]
**F17c-R**	CAACTAACGGGATGTACAGTTTC			
**LT-F**	GCACACGGAGCTCCTCAGTC	218	*ltII*	[53]
**LT-R**	TCCTTCATCCTTTCAATGGCTTT			
**ST-F**	AGGAACGTACATCATTGCCC	521	*st*	[54]
**ST-R**	CAAAGCATGCTCCAGCACTA			
**O26-F**	CAG AAT GGT TAT GCT ACT GT	423	*wzx*	[55]
**O26-R**	CTT ACA TTT GTT TTC GGC ATC			
**O103-F**	TTGGAGCGTTAACTGGACCT	321	*wzx*	[55]
**O103-R**	GCTCCCGAGCACGTATAAG			
**O111-F**	TAG AGA AAT TAT CAA GTT AGT TCC	406	*wzx*	[55]
**O111-R**	ATA GTT ATG AAC ATC TTG TTT AGC			
**O145-F**	CCATCAACAGATTTAGGAGTG	609	*wzx*	[55]
**O145-R**	TTTCTACCGCGAATCTATC			
**O157-F**	CGG ACA TCC ATG TGA TAT GG	259	*wzx*	[55]
**O157-R**	TTG CCT ATG TAC AGC TAA TCC			
**O45-F**	CCG GGT TTC GAT TTG TGA AGG TTG	527	*wzx1*	[56]
**O45-R**	CAC AAC AGC CAC TAC TAG GCA GAA			
**O91-F**	GCTGACCTTCATGATCTGTTGA	291	*gnd*	[57]
**O91-R**	TAATTTAACCCGTAGAATCGCTGC			
**O113-F**	GGGTTAGATGGAGCGCTATTGAGA	771	*wzx*	[12]
**O113-R**	AGGTCACCCTCTGAATTATGGCAG			
**O121-F**	TGGCTAGTGGCATTCTGATG	322	*wzx*	[58]
**O121-R**	TGATACTTTAGCCGCCCTTG			
**O128-F**	GCTTTCTGCCGATATTTGGC		*galF*	[59]
**O128-R**	CCGACGGACTGATGCCGGTGATT			
***aadA1-F***	TATCCAGCTAAGCGCGAACT	447	Streptomycin resistance	[60]
***aadA1-R***	ATTTGCCGACTACCTTGGTC			
***tetA-*** **F**	GGTTCACTCGAACGACGTCA	577	Tetracycline resistance	[60]
***tetA-*** **R**	CTGTCCGACAAGTTGCATGA			
***tetB-*** **F**	CCTCAGCTTCTCAACGCGTG	634	Tetracycline resistance	[60]
***tetB-*** **R**	GCACCTTGCTGATGACTCTT			
***dfrA1-*** **F**	GGAGTGCCAAAGGTGAACAGC	367	Trimethoprim resistance	[61]
***dfrA1-*** **R**	GAGGCGAAGTCTTGGGTAAAAAC			
***Qnr-*** **F**	GGGTATGGATATTATTGATAAAG	670	Fluoroquinolone resistance	[62]
***Qnr-*** **R**	CTAATCCGGCAGCACTATTTA			
***aac*** **[3]** ***-IV-*** **F**	CTTCAGGATGGCAAGTTGGT	286	Gentamicin resistance	[63]
***aac*** **[3]** ***-IV-*** **R**	(TCATCTCGTTCTCCGCTCAT			
***Sul1-*** **F**	TTCGGCATTCTGAATCTCAC	822	Sulfonamide resistance	[63]
***Sul1-*** **R**	ATGATCTAACCCTCGGTCTC			
***blaSHV-*** **F**	TCGCCTGTGTATTATCTCCC	768	Cephalothin resistance	[63]
***blaSHV-*** **R**	CGCAGATAAATCACCACAATG			
***CITM-*** **F**	TGGCCAGAACTGACAGGCAAA	462	Ampicillin resistance	[63]
***CITM-*** **R**	TTTCTCCTGAACGTGGCTGGC			
***ereA-*** **F**	GCCGGTGCTCATGAACTTGAG	419	Erythromycin resistance	[63]
***ereA-*** **R**	CGACTCTATTCGATCAGAGGC			
***cat1-*** **F**	AGTTGCTCAATGTACCTATAACC	547	Chloramphenicol resistance	[63]
***cat1-*** **R**	TTGTAATTCATTAAGCATTCTGCC			
***cmlA-F***	CCGCCACGGTGTTGTTGTTATC	698	Chloramphenicol resistance	[63]
***cmlA-R***	CACCTTGCCTGCCCATCATTAG

**Table 8:** **PCR programs (temperature and volume) for detection of STEC O-serogroups, virulence factors and antibiotic resistance genes**

Gene	PCR program	PCR volume (50 μL)
**O157, O145, O103, O26, O111**	1 cycle:	
	95°C ------------ 3 min.	5 μL PCR buffer 10X
	30 cycle:	1.5 mM Mgcl_2_
	95°C ------------ 20 s	200 μM dNTP^e^
	58°C ------------ 40 s	0.5 μM of each primers F & R
	72°C ------------ 30 s	1.25 U Taq DNA polymerase^e^
	1 cycle:	2.5 μL DNA template
	72°C ------------ 8 min	
**O91, O128, O121, O113, O45**	1 cycle:	
	94°C ------------ 6 min.	5 μL PCR buffer 10X
	34 cycle:	2 mM Mgcl_2_
	95°C ------------ 50 s	150 μM dNTP
	58°C ------------ 70 s	0.75 μM of each primers F & R
	72°C ------------ 55 s	1.5 U Taq DNA polymerase
	1 cycle:	3 μL DNA template
	72°C ------------ 10 min	
***LT, ST***	1 cycle:	
	94°C ------------ 6 min.	5 μL PCR buffer 10X
	34 cycle:	1.5 mM Mgcl_2_
	95°C ------------ 50 s	200 μM dNTP
	56°C ------------ 70 s	0.5 μM of each primers F & R
	72°C ------------ 50 s	1 U Taq DNA polymerase
	1 cycle:	4 μL DNA template
	72°C ------------ 6 min	
***stx1, stx2, eae, hly, cnf1, cnf2, cdtIII, f17c***	1 cycle:	
	95°C ------------ 3 min.	5 μL PCR buffer 10X
	34 cycle:	2 mM Mgcl_2_
	94°C ------------ 60 s	200 μM dNTP
	56°C ------------ 45 s	0.5 μM of each primers F & R
	72°C ------------ 60 s	1.5 U Taq DNA polymerase
	1 cycle:	5 μL DNA template
	72°C ------------ 10 min	
***aadA1, tetA, tetB, dfrA1, qnr, Sul1, aac***[3]***-IV, blaSHV, CITM, cat1, cmlA***	1 cycle:	
	94°C ------------ 8 min.	5 μL PCR buffer 10X
	32 cycle:	2.5 mM Mgcl_2_
	95°C ------------ 60 s	200 μM dNTP
	55°C ------------ 70 s	0.5 μM of each primers F & R
	72°C ------------ 2 min	2 U Taq DNA polymerase
	1 cycle:	3 μL DNA template
	72°C ------------ 8 min	

### Antibiotic susceptibility testing

The Kirby–Bauer disc diffusion method based on the laboratory protocol of the Clinical and Laboratory Standards Institute [64] was used for study the antibiotic susceptibility pattern of *E. coli* isolates. All isolates were cultured on Mueller–Hinton agar in an aerobic condition (Merck, Germany) (24 h at 37°C). Susceptibility of *E. coli* isolates to commonly used antibiotic agents was measured and interpreted based on the CLSI protocol. *E. coli* ATCC 25922 was used as quality control.

### Statistical analysis

The SPSS (Statistical Package for the Social Sciences) software (Ver. 16) and Chi-square and Fisher's exact tests were used in order to study the statistical relationship between the incidence of bacterium in various age, season and geographical regions and also between the frequency of various virulence factors, pathotypes, antibiotic resistance genes and serogroups. A *P* value < 0.05 was considered statistically significant.
